# Delineating significant genome-wide associations of variants with antipsychotic and antidepressant treatment response: implications for clinical pharmacogenomics

**DOI:** 10.1186/s40246-019-0254-y

**Published:** 2020-01-15

**Authors:** Maria Koromina, Stefania Koutsilieri, George P. Patrinos

**Affiliations:** 1 Laboratory of Pharmacogenomics and Individualized Therapy, Department of Pharmacy, School of Health Sciences, University of Patras, University Campus, Rion, GR-265 04 Patras, Greece; 2 Department of Pathology, College of Medicine and Health Sciences, United Arab Emirates University, Al-Ain, United Arab Emirates; 3 Zayed Center of Health Sciences, United Arab Emirates University, Al-Ain, United Arab Emirates

**Keywords:** Antipsychotics, Antidepressants, Pharmacogenomics, Statistical assessment, GWAS catalog, GWAS findings, Clinically approved guidelines

## Abstract

**Background:**

Genome-wide association studies (GWAS) have significantly contributed to the association of many clinical conditions and phenotypic characteristics with genomic variants. The majority of these genomic findings have been deposited to the GWAS catalog. So far, findings uncovering associations of single nucleotide polymorphisms (SNPs) with treatment efficacy in mood disorders are encouraging, but not adequate.

**Methods:**

Statistical, genomic, and literature information was retrieved from EBI’s GWAS catalog, while we also searched for potential clinical information/clinical guidelines in well-established pharmacogenomics databases regarding the assessed drug-SNP correlations of the present study.

**Results:**

Here, we provide an overview of significant genome-wide associations of SNPs with the response to commonly prescribed antipsychotics and antidepressants. Up to date, this is the first study providing novel insight in previously reported pharmacogenomics associations for antipsychotic/antidepressant treatment. We also show that although there are published CPIC guidelines for antidepressant agents, as well as the FDA labels include genome-based drug prescription information for both antipsychotic and antidepressant treatments, there are no specific clinical guidelines for the assessed drug-SNP correlations of this study.

**Conclusions:**

Our present findings suggest that more effort should be implemented towards identifying GWA-significant antipsychotic and antidepressant pharmacogenomics correlations. Moreover, additional functional studies are required in order to characterise the potential role of the assessed SNPs as biomarkers for the response of patients to antipsychotic/antidepressant treatment.

## Background

Pharmacogenomics refers to the use of genomic biomarkers to predict an individual’s response to drug efficacy and toxicity. Pharmacogenomics has also seen a raise in terms of research findings, which span over the last decade. Although additional clinical factors such as disease severity, diet, and concurrent medications clearly contribute to the variability in response to drug therapy, inherited differences in the metabolism and action of drugs at their target sites or in the pharmacokinetics and pharmacodynamics of a drug harbor a predominant effect in the therapy outcome [1, 2]. The complexity of drug response can be multifactorial and variable over time, since subjective clinical scales are usually implemented, thus making it challenging to identify genetic variants that robustly predict drug response.

Over the last decade, genome-wide association studies (GWAS) have widely focused on tailoring the genetic background of psychiatric diseases [3, 4]. However, most of these findings usually fail to be replicated in subsequent genetic studies. Candidate gene studies have also been performed as an alternative approach, although the sample sizes have been quite small in some instances [5]. Regardless of each study’s design limitations, genetic studies have provided useful insight in delineating associations of genetic variation with psychiatric disease [6–8] and new ideas about disease etiology. Interesting psychiatric genetic findings include specific SNPs, which were characterised as genome-wide significant for both bipolar disorder and schizophrenia. These SNPs were identified within the following genes: *CACNA1CS, ANK3*, and *ITIH3-ITIH4*. In contrast, SNPs within *MHC, ODZ4*, *TCF4*, and other genetic loci were genome-wide significant for either disorder separately but not for both [9].

GWAS for delineating drug treatment response and toxicity for psychiatric disorders have also been performed in order to tailor antipsychotic or antidepressant treatment. It is worth noting that most of the identified associations have not been individually replicated, thus leaving the pharmacogenomics background of commonly prescribed antipsychotic or antidepressant drugs quite vague. To this end, Allen and Bishop performed a systematic review of the existing literature for GWAS findings for antipsychotic treatment response. In this review, 15 genome-wide significant loci were identified (*CNTNAP5*, *GRID2*, *GRM7*, 8q24 *(KCNK9)*, *PCDH7*, *SLC1A1* and *TNIK)*, seven of which were replicated in other antipsychotic genome-wide studies [10]. However, further validation of these findings is needed in order to demonstrate the clinical utility of these pharmacogenomics markers.

The United States Food and Drug Administration (FDA; http://www.fda.gov) started incorporating pharmacogenomics information on its labels especially after 2005 and the completion of the Human Genome Project (“www.fda.gov”, [11]). Almost 15 years later, more than 200 drug labels are accompanied by pharmacogenomics information highlighting the progressive acknowledgment of the major regulatory body regarding this field of precision medicine [12]. Such information is gathered and represented in detail in the FDA’s Table of Pharmacogenomics Biomarkers in Drug Labels [13] bearing variable levels of significance, from informational to necessary guidance.

The unmet need, for translating the voluminous literature into the association pairs that could serve as possible biomarkers in a real-time clinical setting, was significantly catalyzed by the formation of the renowned online resource, the Clinical Pharmacogenetics Implementation Consortium (CPIC; http://www.cpicpgx.org) in late 2009 [49, 50]. So far, the CPIC has published 23 peer-reviewed and evidence-based guidelines for over 60 drug–gene pairs in an effort to bridge the gap between the available pharmacogenomics test results and their usefulness in genome-informed prescribing [15]. As for the drugs implicated in antidepressant treatment, two guidelines regarding the selective serotonin reuptake inhibitors (SSRI’s) and the tricyclic antidepressants (TCA’s) and their association with *CYP2D6* and *CYP2C19* were published in August 2015 and December 2016, respectively [54, 55].

In this study, we aimed to identify and assess genetic associations either with GWA or with nominal significance for commonly prescribed antipsychotic and antidepressant treatment, for which clinical guidelines exist. For this purpose, we assessed the pharmacogenomics associations of commonly prescribed antidepressant/antipsychotic drugs as these were reported in publications deposited in the EBI-GWAS catalog.

## Results

### Assessing the GWAS catalog data for identification of psychiatric pharmacogenomics findings

First, we began by downloading the pharmacogenomics GWA findings from 20 research studies and by excluding review papers (Additional file 1: Table S1). We extracted the following information from each study: the assessed drug (type of antidepressant or antipsychotic compound), the SNP associated with drug response, the gene within which we identified the specific SNP, the PubMed ID of the study, as well as the web link.

Subsequently, we performed literature search in order to annotate the study type of each one of the 20 research studies. The study findings could be either GWAS findings, or (the findings) could constitute a combination of findings from GWAS and (genome-wide) cell assays. We also identified SNPs, which were associated with response to more than one drug related to antipsychotic or antidepressant treatment (Additional file 1: Table S1). More precisely, 555 drug–SNP correlations were identified in total, of which 462 drug–SNP correlations were characterised as unique (Additional file 1: Table S2) upon removal of any duplicate drug–SNP correlations.

**Fig. 1:**
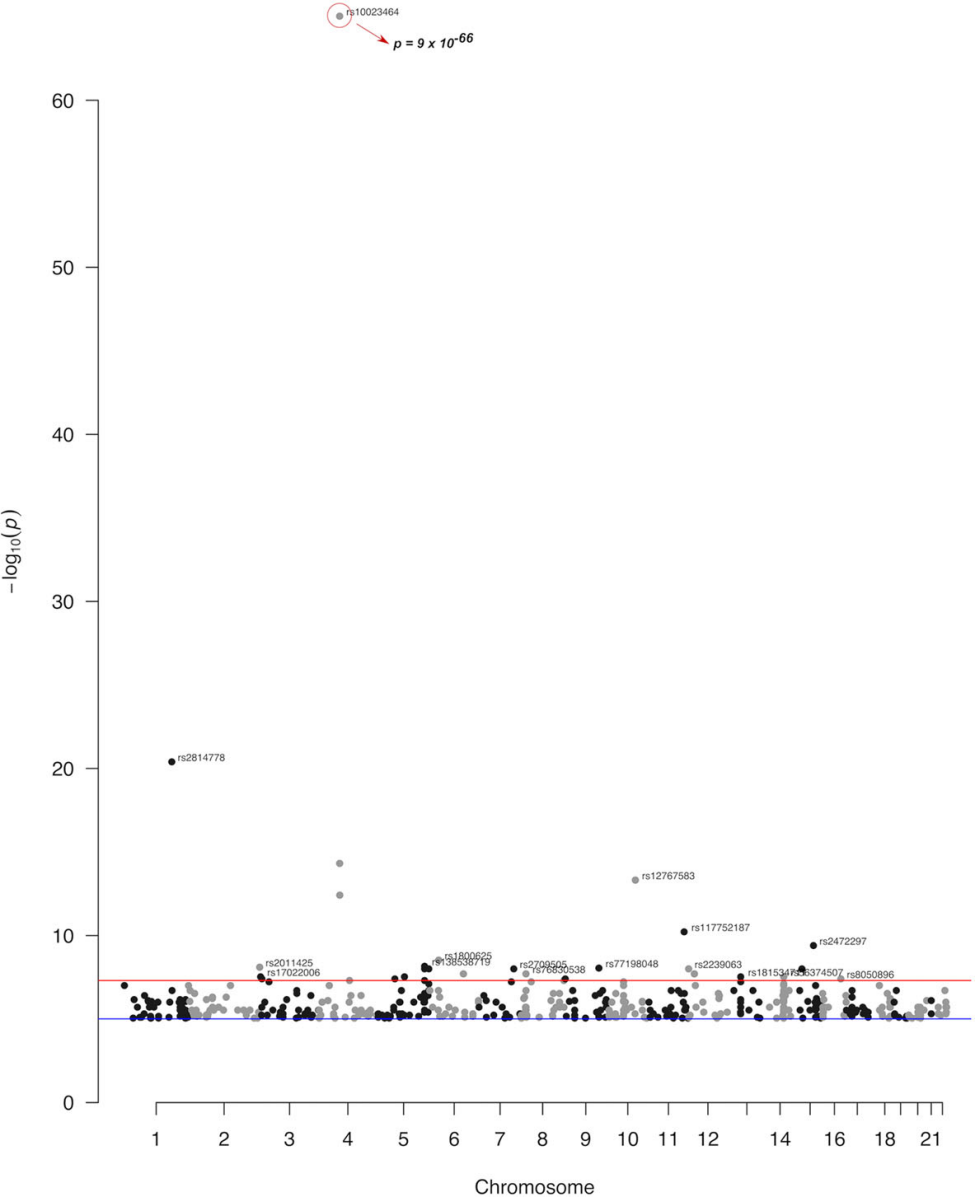
Manhattan plot of the significant associations of pharmacogenomics variants as identified in the GWAS studies deposited in the GWAS catalog. These variants have been associated with response to antipsychotic treatment. The red line denotes the genome-wide threshold of significance (*p* = 5× 10^-8^) and the blue line the suggestive threshold of significance (*p* = 10^-5^)

Manhattan plots were also created in order to highlight the significance levels of the SNP associations with antipsychotic or antidepressant drug response. Interestingly, one of these variants had a GWA *p* value exceeding the level of GWA significance (*p* = 9 × 10^-66^). This variant was rs10023464 SNP (*AC093720.1* - *AC021146.8* intergenic region) that was characterised as a locus associated with plasma clozapine–norclozapine ratio in treatment-resistant schizophrenia patients. Amongst SNPs, which exceeded the GWA significance level, are rs11725502 (*AC021146.8* - *UGT2B10* intergenic region; *p* = 5 × 10^-15^), rs12767583 (*CYP2C19*; *p* = 5 × 10^-14^), rs7668556 (*AC111000.6* - *AC111000.1* intergenic region; *p* = 4 × 10^-13^), rs2814778 (*ACKR1*; *p* = 4 × 10^-21^), and rs117752187 (*MIR100HG*; *p* = 6 × 10^-11^). These SNPs were associated with clozapine response, except for rs117752187, which was associated with paliperidone response (Fig. 1).

**Fig. 2:**
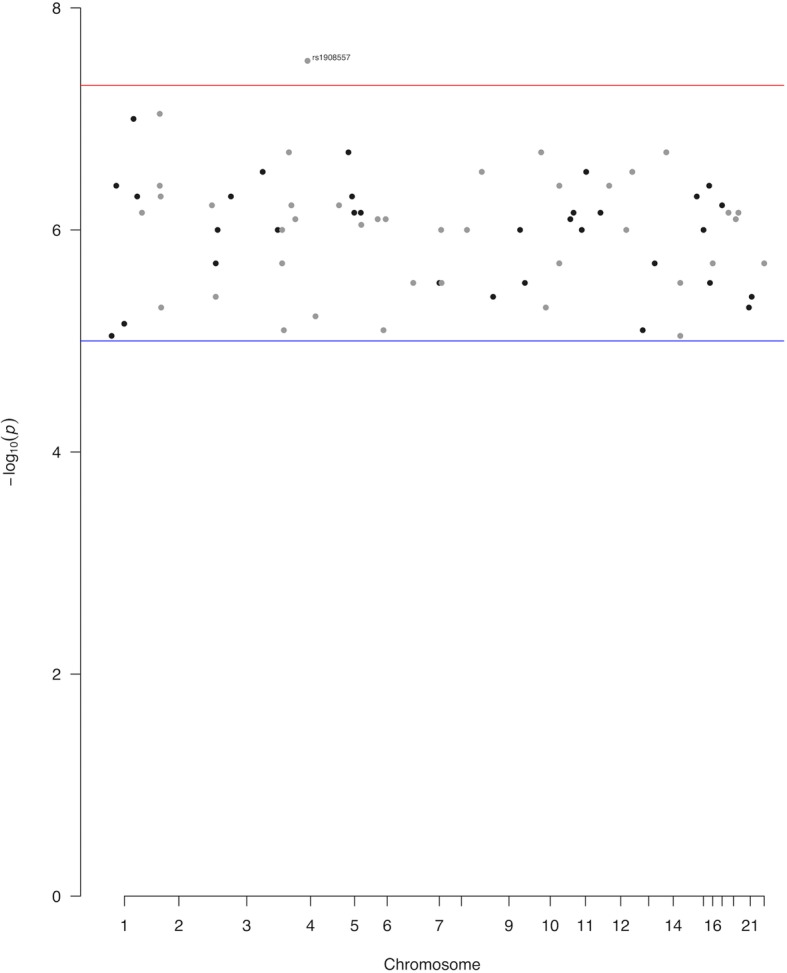
Manhattan plot of the significant associations of pharmacogenomics variants as identified in the GWAS studies deposited in the GWAS catalog. These variants have been associated with response to antidepressant treatment. The red line denotes the genome-wide threshold of significance (*p* = 5 × 10^-8^) and the blue line the suggestive threshold of significance (*p* = 10^-5^)

Another interesting observation from the Manhattan plots is the higher number of drug–SNP correlations for antipsychotic drugs compared to antidepressant treatment (Fig. 2). However, this observation could be explained by the overall limited number of the assessed GWAS studies.

**Fig. 3:**
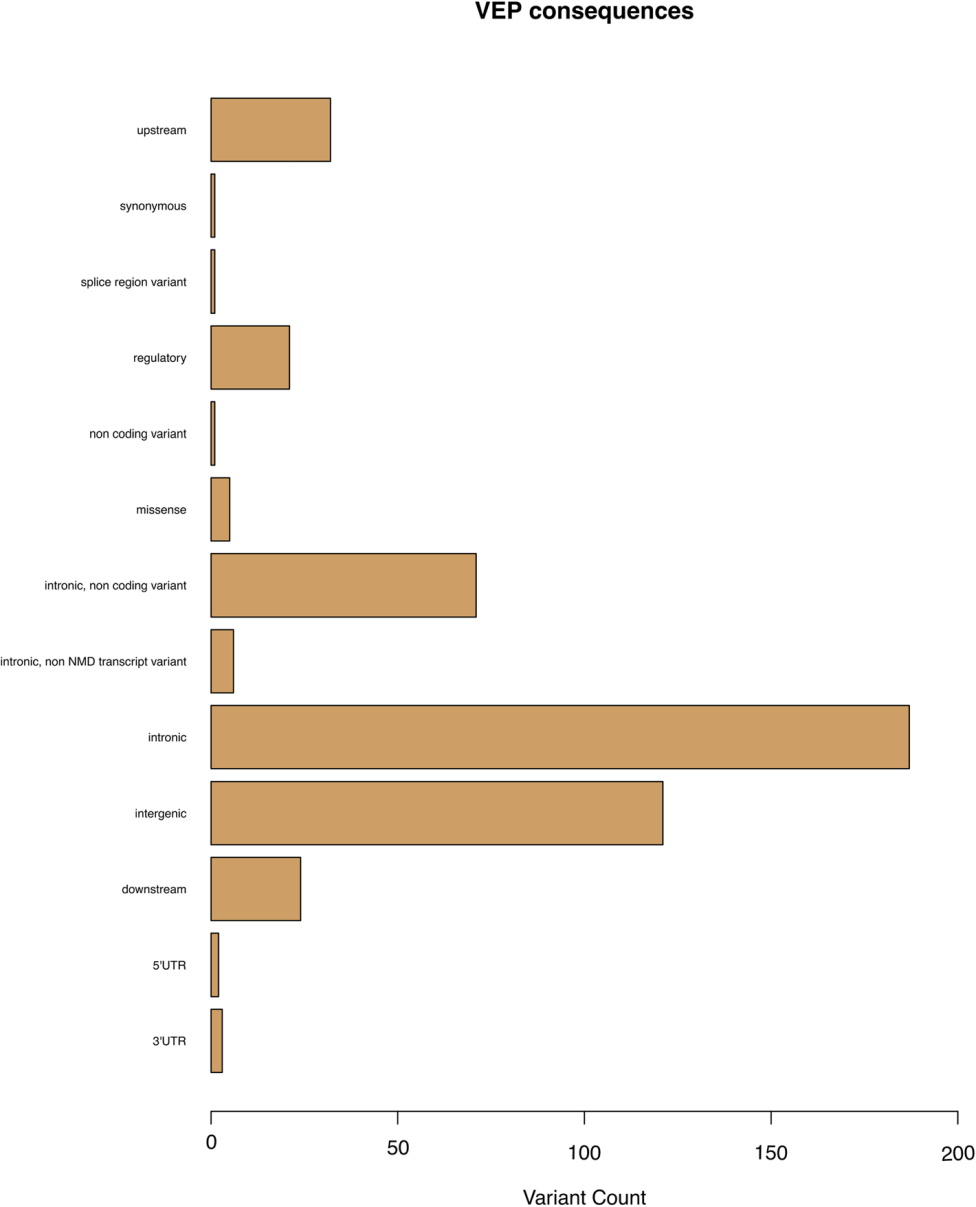
Plot of the number of pharmacogenomics variants assessed in the present study against their Variant Effect Prediction (VEP) consequence. NMD, nonsense mediated decay; 5’UTR, variant in the 5’UTR region; 3’UTR, variant in the 3’UTR region

Regarding the SNPs’ functional protein consequence, more than 90% of the identified GWA-significant SNPs were characterised as intronic or intergenic variants, whilst only 5 were characterised as missense: rs2236295, rs2307441, rs17815774, rs17727261, and rs41314643 (Table 1; Fig. 3). rs2236295 (*ADO*) and rs2307441 (*POLG*) were identified within the first genome-wide association study (GWAS) in Generalized Anxiety Disorder (GAD), and they were characterised as potential predictors of venlafaxine extended release (XR) treatment outcome. Moreover, rs17815774 (*TRPM1*) and rs17727261 (*CNTNAP5*) were identified in a genome-wide pharmacogenomics study and they were associated with the response to treatment with risperidone. Lastly, in another GWAS study, rs41314643 (*NMNAT2*) was associated with the risk of clozapine-induced agranulocytosis/granulocytopenia.

**Table 1:** Functional annotation of the five identified missense variants within the psychiatric pharmacogenomics GWAS studies of interest in this study

Variant type	rsID	Gene	Drug correlation	Protein damaging pred. (SIFT^*^, Polyphen2^#^)
missense	rs4314643	*NMNAT2*	Clozapine	Benign (0.13, 0.232)
missense	rs2236295	*ADO*	Venflaxine	Benign (0.74, 0.007)
missense	rs17815774	*TRPM1*	Risperidone	Benign (0.07, 0.191)
missense	rs2307441	*POLG*	Venlafaxine	Benign (0.16, 0.334)
missense	rs17727261	*CNTNAP5*	Risperidone	Benign (0.06, 0.006)

SIFT and Polyphen2 are *in silico* tools used to assess the protein damaging effect of missense variants

*SIFT score for protein damaging prediction (< 0.05; deleterious)

^#^Polyphen2 score for deleterious variants ( > 0.908; probably damaging, 0.446 < score ≤ 0.908; possibly damaging)

Μoreover, we assessed the number of drug–SNP correlations, after removing any duplicate associations, and we observed a high number of common SNPs associated with antidepressant or antipsychotic efficacy and toxicity (Table 2). An almost equal number of low frequency, rare, and SNPs with no 1000 Genomes MAF was also observed, whilst the lowest number of drug-SNP correlations was found within SNPs with intermediate frequency (Table 2). This observation indicates that a couple of the identified drug-common SNP associations could be correlated with potential clinical actionability of these SNPs.

**Table 2:** Number of common (MAF > 0.10), intermediate frequency (MAF 0.05–0.10), low frequency (0.01–0.05) and rare variants (MAF < 0.01) for each assessed drug

Drug	Common (*N* genes or SNPs)	Interm (*N* genes or SNPs)	Low freq (*N* genes or SNPs)	Rare (*N* genes or SNPs)	No1000 G MAF (*N* genes or SNPs)	Total (per drug)
Aripiprazole	2	-	-	-	-	2
Bupropion	6	-	5	8	2	21
Clozapine	35	7	16	11	8	77
Citalopram	7	2	7	8	7	31
Duloxetine	1	-	-	-	1	2
Escitalopram	3	1	1	8	4	17
Haloperidol	6	1	-	-	1	8
Iloperidone	5	1	-	-	-	6
Ketamine	13	2	-	-	4	19
Lurasidone	11	-	-	-	2	13
Olanzapine	10	3	-	-	2	15
Oxcarbazepine	4	1	2	-	-	7
Paliperidone	56	22	34	56	24	192
Perphenazine	11	1	3	-	1	16
Quetiapine	14	1	1	-	2	18
Risperidone	15	-	1	-	4	20
Sertraline	-	1	1	-	-	2
Venlafaxine	11	1	2	1	3	18
Ziprasidone	8	1	-	1	-	10
Total (per MAF)	218	45	73	93	65	

The number of SNPs counted is equal to the number of genes as duplicates of the same drug–SNP correlations were removed

*interm* intermediate, *low freq* low frequency, *MAF* minor allele frequency

### Measuring the association strength of the identified SNPs based on their allele frequency

**Fig. 4:**
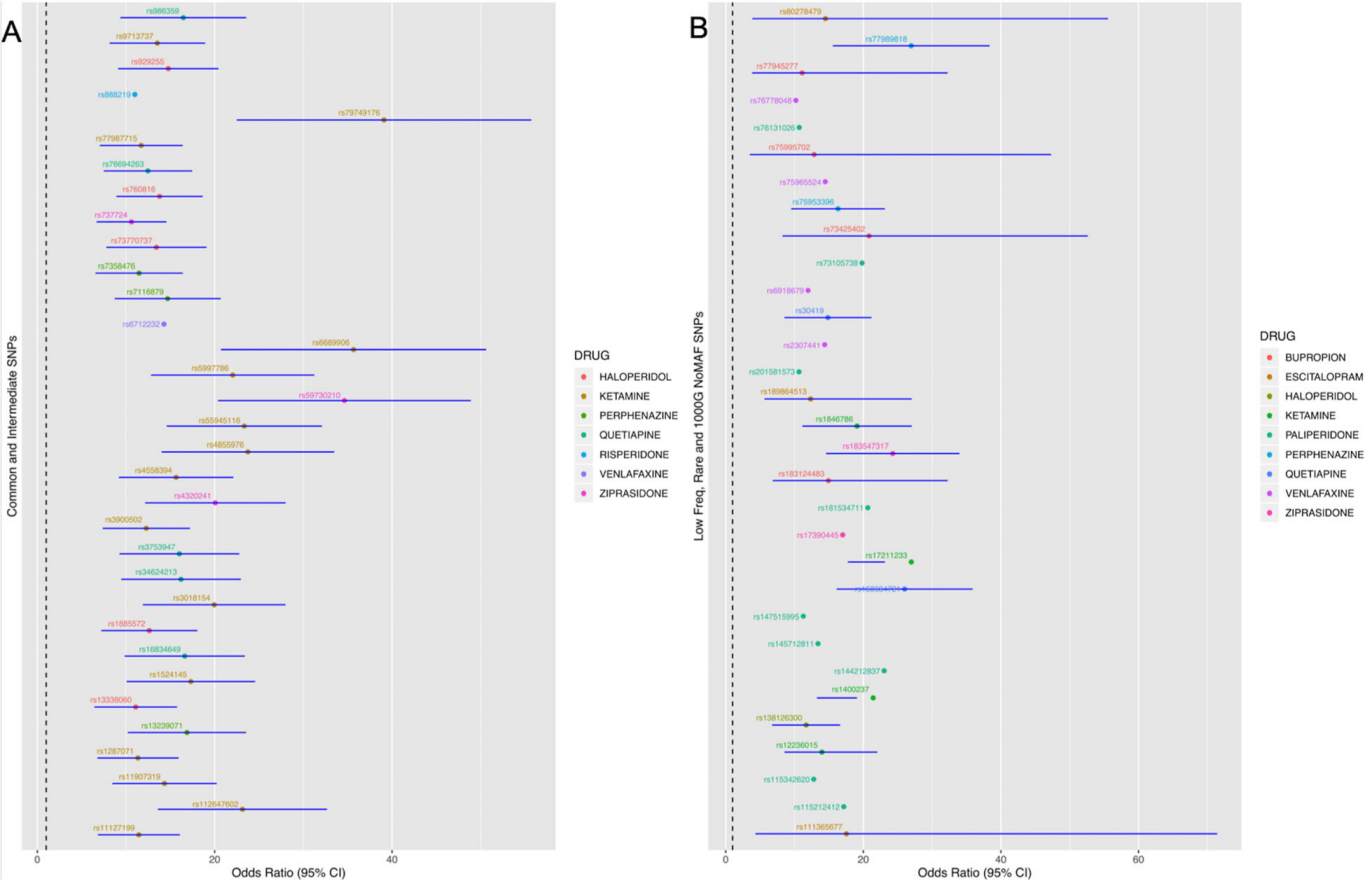
**a** Odds ratio values of pharmacogenomics (PGx) variants of common (MAF > 0.10) or intermediate (0.05 ≤ MAF ≤ 0.10) frequency are plotted alongside with the 95% of their confidence intervals. The PGx variants are colored based on the antipsychotic or antidepressant drug of the association. **b** Odds ratio values of pharmacogenomics (PGx) variants of low frequency (0.01 ≤ MAF < 0.05); rare (MAF < 0.01) frequency are plotted alongside with the 95% of their confidence intervals. Moreover, the odds ratio values and the confidence intervals of pharmacogenomics variants that were not found within the 1000 Genomes project are also plotted. The PGx variants are colored based on the antipsychotic or antidepressant drug association

As explained in the Methods section, odds ratio values (ORs) and confidence interval values (CIs) were retrieved and assessed for the identified SNPs (Fig. 4). These values provide an estimation for the protective or risk effect of these SNPs and can be directly associated with the potential clinical outcome. More precisely, in this section, we aim to delineate the SNP effect on the risk for showing adverse drug reactions (ADRs) by analysing odds ratio values and their association with ADR risk. The drug–SNP associations with OR higher than 1 were associated with an increased risk for developing ADRs, whilst drug–SNP associations with OR less than 1 were associated with a lower risk for developing ADRs.

Additional data (Additional file 2: Figsure S1–S10) show the ORs and CIs plotted for all identified SNPs and color-coded based on the drug compound. Two graphs, one with OR less than 1 and one with OR bigger than 1, were created for the 5 different classes of MAFs: common (1000 Genomes MAF higher than 10%), intermediate (1000 Genomes MAF between 5 and 10%), low-frequency (1000 Genomes MAF between 1 and 5%), rare (1000 Genomes MAF less than 1%), and finally variants for which there was no MAF in the 1000 Genomes Project.

We observed that there were an equally high number of common SNPs that had ORs either below 1 or above 1, thus hinting at a decreased or increased possibility with drug toxicity, respectively. Similar observations were also applied for GWA-significant drug—intermediate frequency SNP correlations.

In contrast, most of the rare and low-frequency variants had high ORs, usually higher than 5, thus suggesting that these variants can be potentially used for further clinical tests. Our findings indicate that the majority of SNPs with frequency less than 5% have high ORs, thus hinting that these SNPs may be associated with increased drug toxicity. SNPs that have an OR less than 1 indicate an inversed association, which is basically defined as protection against the drug response (Additional file 2: Figures S5–S10). Interestingly, all low-frequency and rare SNPs as well as SNPs that were not annotated in 1000 Genomes, with ORs between 0 and 1, have been associated with reduced probability for drug toxicity.

Amongst the SNPs with high ORs were rs117375960 and rs188843168, which were excluded from the forest plots in order to allow a clearer presentation of the findings. rs117375960 was identified within *NT5C2* and was associated with the response to citalopram and escitalopram (*p* = 2 × 10^-6^, OR = 142.86). Similarly, rs188843168 (*ATP5MD*) was also associated with the response to citalopram and escitalopram (*p* = 4 × 10^-7^, OR = 449.83). Both of these SNPs are associated with an increased risk for drug toxicity induced from citalopram or escitalopram treatment.

Forest plots were also created for variants that had ORs higher than 10. As also stated in the Methods section, this OR cut-off value was selected since the majority of the clinical studies suggest an OR greater than 10, as sufficient enough for decision-making. However, since the ORs for most of these variants were quite high, it was difficult to provide an accurate estimate of the confidence intervals for these variants. The high ORs combined with the statistically significant association of the identified SNPs provide evidence for the potential role of these variants as biomarkers for antipsychotic drug toxicity.

### Lack of CPIC guidelines for the presented antipsychotic drug–SNP correlations

In an effort to mine valuable pharmacogenomics information from the CPIC online resource, we found out that the commonly prescribed antipsychotic agents, namely clozapine, risperidone, haloperidol, perphenazine, aripiprazole, iloperidone, and olanzapine, are not yet accompanied by a corresponding pharmacogenomics (PGx) guideline. However, all the aforementioned drugs are associated with *CYP2D6* bearing a CPIC level B or C, while their corresponding CPIC guideline will be compiled in the future. Of note, the US regulatory body already includes pharmacogenomics information regarding *CYP2D6* in the respective drug labels, either vaguely commenting on the pharmacokinetics of the administered agent (as in the case of risperidone-*CYP2D6*) or proposing specific genome-informed dose adjustment (as in the case of aripiprazole-*CYP2D6*). Two SNRI’s, venlafaxine and duloxetine, are deemed to be accompanied by a CPIC guideline in the future regarding their association with *CYP2D6*. As for the SSRI’s, citalopram, escitalopram, and sertraline, a published CPIC guideline proposes specific therapeutic strategies based on *CYP2C19* genotype results. In parallel, the US regulatory body refers to the pharmacogenomics information related to the aforementioned drug–gene association pairs in the corresponding drug inserts. It is noteworthy that there is no mention regarding the variants discussed in our paper that are deemed ambitious as for their clinical actionability in either two sources.

## Discussion

There is no doubt that GWAS research has yielded many discoveries in the field of neuropsychiatric and neurodevelopmental diseases. However, constant research and improvements in “big data” parsing methods are essential for improvements in the discovery of pharmacogenomics findings. GWAS studies can be useful in unraveling potential mechanisms and pathways that underlie human characteristics, diseases, and drug response.

For example, researchers in the Schizophrenia Working Group of the Psychiatric Genetics Consortium (PGC) performed a GWAS and identified 108 genome-wide significant loci [18], thus showing that increased sample sizes can boost the discovery in schizophrenia genetics research and psychiatric pharmacogenomics research.

As with most GWAS studies, those focusing on identification of drug-genomic variant correlations may be characterised by heterogeneity issues across the different study sample datasets. Any discrepancies between individual studies must be taken into consideration as confounding variables. For example, studies included a broad range of sample sizes, some of which only included a single racial or ethnic group; these studies should be replicated within different populations [51].

Nonetheless, PGx GWAS have resulted in the identification of several actionable genetic variants that have been genotyped and used to inform drug selection and dosage. However, the most significant PGx GWAS achievements have been associated with the identification of non-HLA markers, such as *NUDT15*, which is associated with thiopurine-induced leukopenia [19].

So far, only a few psychiatric clinical pharmacogenomics and psychiatric PGx GWAS studies have been published thus pointing to a need for properly designed psychiatric pharmacogenomics. Two characteristic examples of clinical psychiatric PGx studies, which are currently on-going, are the following: one is the “PREPARE” by the Ubiquitous Pharmacogenomics (U-PGx; http://www.upgx.eu) Consortium and the other one is an effort run at the Karolinska Institute [20, 21]. The “PREPARE” study aims to perform pre-emptive genotyping of a panel of clinically relevant PGx-markers, for which guidelines are available, whilst being implemented across healthcare institutions in seven European countries. Amongst the individuals recruited are individuals diagnosed with a variety of psychiatric disorders and the upper study goal is the identification of biomarkers associated with antipsychotic/antidepressant drug responses [21]. Regarding the other effort, as run by the Karolinska Institute, findings from this study indicate that *CYP2D6* pre-emptive genotyping would be valuable for individualising risperidone and aripiprazole dosing, thus leading to treatment optimisation [20].

It is worth to note that only about half of the psychiatric genome-wide significant associations have been validated in subsequent studies, and more precisely either in an independent study or in a replication sample. Because of the possibility of false discovery, the likelihood of a GWAS signal being a true marker of the tested phenotype holds fairly limited promise prior to replication. Overall, findings from both of these efforts highlight the need for improving and boosting discovery of clinically relevant psychiatric pharmacogenomics biomarkers [22].

As of submission of this manuscript, this is the first study focusing on 20 published research articles, as deposited in the GWAS catalog, and which assesses GWA-significant antipsychotic/antidepressant drug–SNP correlations. Interestingly, only two SNPs were identified within or between clinically approved pharmacogenes, (rs2472297 between *CYP1A1-CYP1A2* and rs12767583 within *CYP2C19)* and they were both associated with the response to clozapine. No clinical guidelines either from CPIC or from FDA exist for the rest of the identified drug–SNP correlations.

Undoubtedly, implementation of PGx research findings in the clinic requires lots of time and effort owing to the ever-increasing need for functional validation studies, regulatory clearance and development of the appropriate translational tools. To this end, our findings can be proven useful for unravelling the background for identification of pharmacogenomics biomarkers for psychiatric drug treatment. These findings may be proven meaningful for informing the findings from pharmacogenomics clinical studies (i.e., Ubiquitous Pharmacogenomics—U-PGx), since lots of the assessed antipsychotic/antidepressant compounds are included in the U-PGx clinical study. Therefore, the identified drug–SNP correlations may be of particular interest in future pharmacogenomics clinical studies and they may be in linkage disequilibrium with other known psychiatric pharmacogenomics biomarkers.

## Conclusions

To our knowledge, this is the first study that summarizes and provides novel insight in previously identified and GWA-significant pharmacogenomics associations for antipsychotic/antidepressant treatment. We showed that common pharmacogenomics association for antipsychotic/antidepressant drugs with genomic biomarkers might harbour large effect sizes, thus hinting at a potential clinical utility. We also demonstrate that there are multiple drug–SNP correlations, for which little or no clinical information is available and for which no approved clinical guidelines exist. So far, little is known about the functional impact of variants on inter-individual variability in drug response, which are identified in genomic loci apart from well-characterised pharmacogenes. Το this end, more effort should be placed towards the identification of pharmacogenomics biomarkers for antipsychotic treatments. This can be achieved by leveraging the information from databases, such as the GWAS catalog, as well as by updating and renewing the freely available information regarding clinically approved guidelines (i.e. CPIC, FDA).

## Methods

### Data collection

The data of this study include all studies recorded by the GWAS catalog [23] with the date of the datasets download being the 27th of August 2019. Statistical, genomic, and literature information were retrieved from 20 research articles (Additional file 1: Table S1) [4, 24–46] as deposited in the GWAS catalog of EBI. The information of the datasets includes literature sources, phenotype information, *p* values, and identified SNPs, ORs and CIs.

The selection of antipsychotic or antidepressant drugs was based on examples of antidepressant or antipsychotic medications, which are the gold-standard treatment options for schizophrenia and other common mental conditions. The query criteria in the GWAS catalog database were the name of the assessed antipsychotic or antidepressant compounds.

### Statistical analysis

Odds ratio values, chi-square *p* values, and 95% of confidence intervals were used in order to statistically examine the association strength of the GWA significant SNPs. These statistical measurements were also directly retrieved from the GWAS catalog. Moreover, drug–SNP correlations were examined in order to identify potential associations, which were identified in more than one study. The results were presented either as Forest plots or Manhattan plots and they were created in R software (“https://www.r-project.org” [46–48]). Forest plots were built in the ‘ggplot2’ R package, while Manhattan plots were created by implementing the ‘qqman’ R package [16, 17]. Moreover, forest plots were also created separately for SNPs with ORs higher than 10, since most studies suggest 10 as a threshold sufficient enough for decision-making [52].

### Variant annotation

Functional annotation of the identified SNPs and MAF annotation from the 1000 Genomes Project was performed using the Variant Effect Predictor (VEP) freely available from ENSEMBL [53].

### PGx information mining

Moreover, specific pharmacogenomics information was retrieved from the FDA’s Table of Pharmacogenomics Biomarkers in Drug Labels regarding the pharmaceutical agents currently used in antidepressant and antipsychotic treatment, as well as from the respective published CPIC guidelines and their supplementary information [54, 55].

## Supplementary information

**Additional file 1: Table S1.** Summary information for the identified drug SNP-correlations. **Table S2.** List of the antipsychotic/antidepressant drug-SNP correlations that have been reported once (duplicates removed) in the assessed 20 studies deposited in the GWAS catalog.

**Additional file 2: Figures S1-S10.** showing the odds ratio values split in two categories (between 0-1 and between 1-60) for commonfrequency (MAF > 0.10), intermediate frequency (0.05 <= MAF <= 0.10), low frequency (0.01 <= MAF < 0.05), rare frequency (MAF < 0.01) variants as well as for variants not found in 1000Genomes Project. The PGx variants are colored based on the antipsychotic or antidepressant drug of the association.

## Data Availability

All datasets are publicly available specified in the manuscript. The initial data, as retrieved from the GWAS catalog, can be found in the Additional file 1.

## Abbreviations

ADRs: Adverse drug reactions
CI: Confidence interval
CPIC: Clinical Pharmacogenetics Implementation Consortium
EBI: European Bioinformatics Institute
FDA: Food and Drug Administration
GAD: Generalised anxiety disorder
GWA: Genome-wide association
GWAS: Genome-wide association study
MAF: Minor allele frequency
OR: Odds ratio
PGC: Psychiatric Genetics Consortium
PGx: Pharmacogenomics
SNP: Single-nucleotide polymorphism
SNRI: Serotonin norepinephrine reuptake inhibitor
SSRI: Selective serotonin reuptake inhibitor
U-PGx: Ubiquitous pharmacogenomics
VEP: Variant effect predictor
XR: Extended release
