# Supplementary material for: Delineating significant genome-wide associations of variants with antipsychotic and antidepressant treatment response: implications for clinical pharmacogenomics
Source: Hum Genomics. 2020 Jan 15;14:4. doi: 10.1186/s40246-019-0254-y (PMC6964087; doi:10.1186/s40246-019-0254-y)
Supplement: Supplementary file 2 — Additional file 2: Figures S1-S10. showing the odds ratio values split in two categories (between 0-1 and between 1-60) for commonfrequency (MAF > 0.10), intermediate frequency (0.05 <= MAF <= 0.10), low frequency (0.01 <= MAF < 0.05), rare frequency (MAF < 0.01) variants as well as for variants not found in 1000Genomes Project. The PGx variants are colored based on the antipsychotic or antidepressant drug of the association. [file 40246_2019_254_MOESM2_ESM.pdf]

Supplementary information

Delineating significant genome-wide associations of variants with antipsychotic and antidepressant treatment response: Implications for clinical pharmacogenomics

Maria Koromina [1], Stefania Koutsilieri [1], George P. Patrinos [1,2,3]

[1] Department of Pharmacy, School of Health Sciences, University of Patras, Patras, Greece;

[2] Department of Pathology, College of Medicine and Health Sciences, United Arab Emirates University, Al-Ain, United Arab Emirates;

[3] Zayed Center of Health Sciences, United Arab Emirates University, Al-Ain, United Arab Emirates

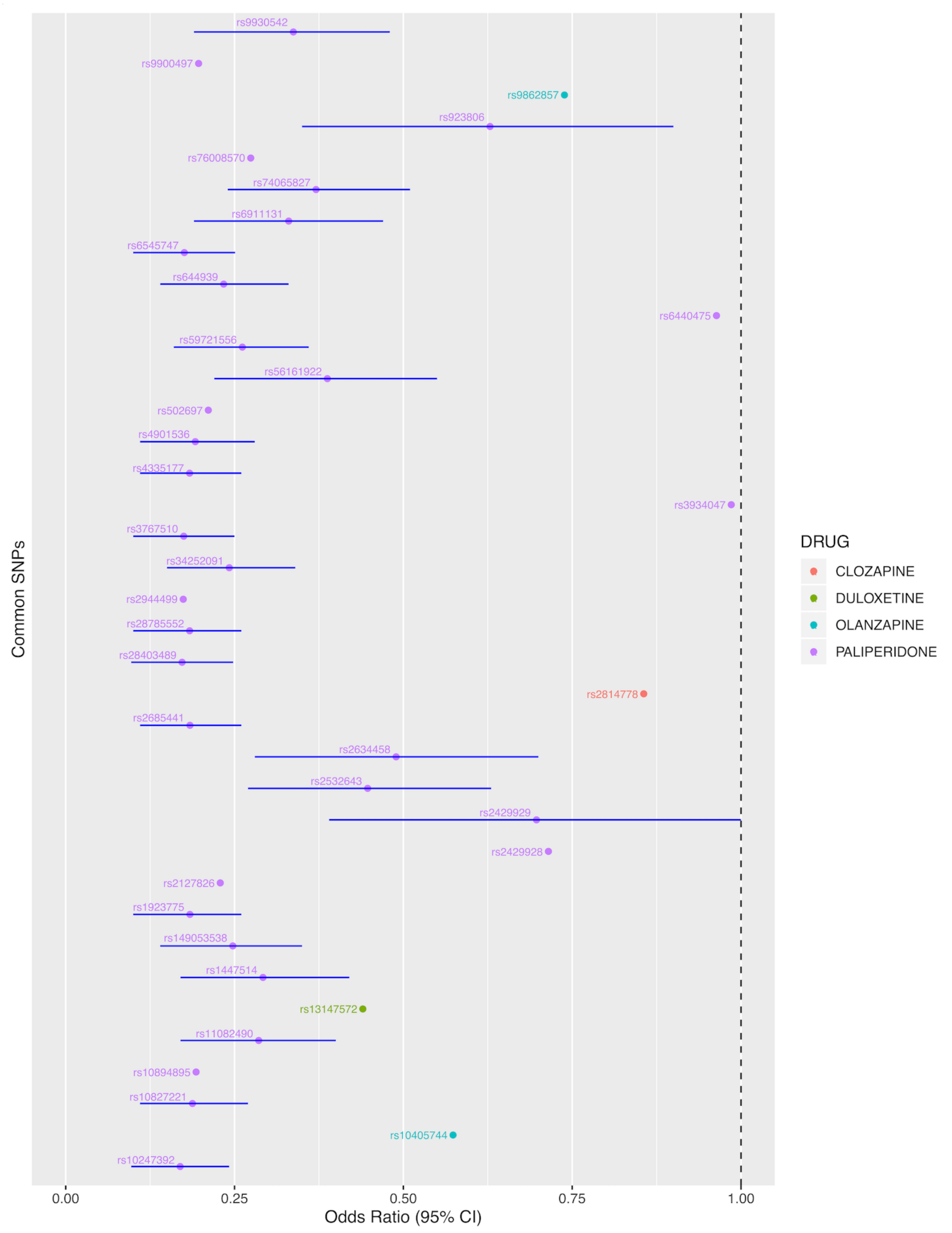

Figure S1. Odds ratio values (between 0-1) of pharmacogenomics (PGx) variants of common frequency (MAF > 0.10) are plotted alongside with the 95% of their confidence intervals. The PGx variants are colored based on the antipsychotic or antidepressant drug of the association.

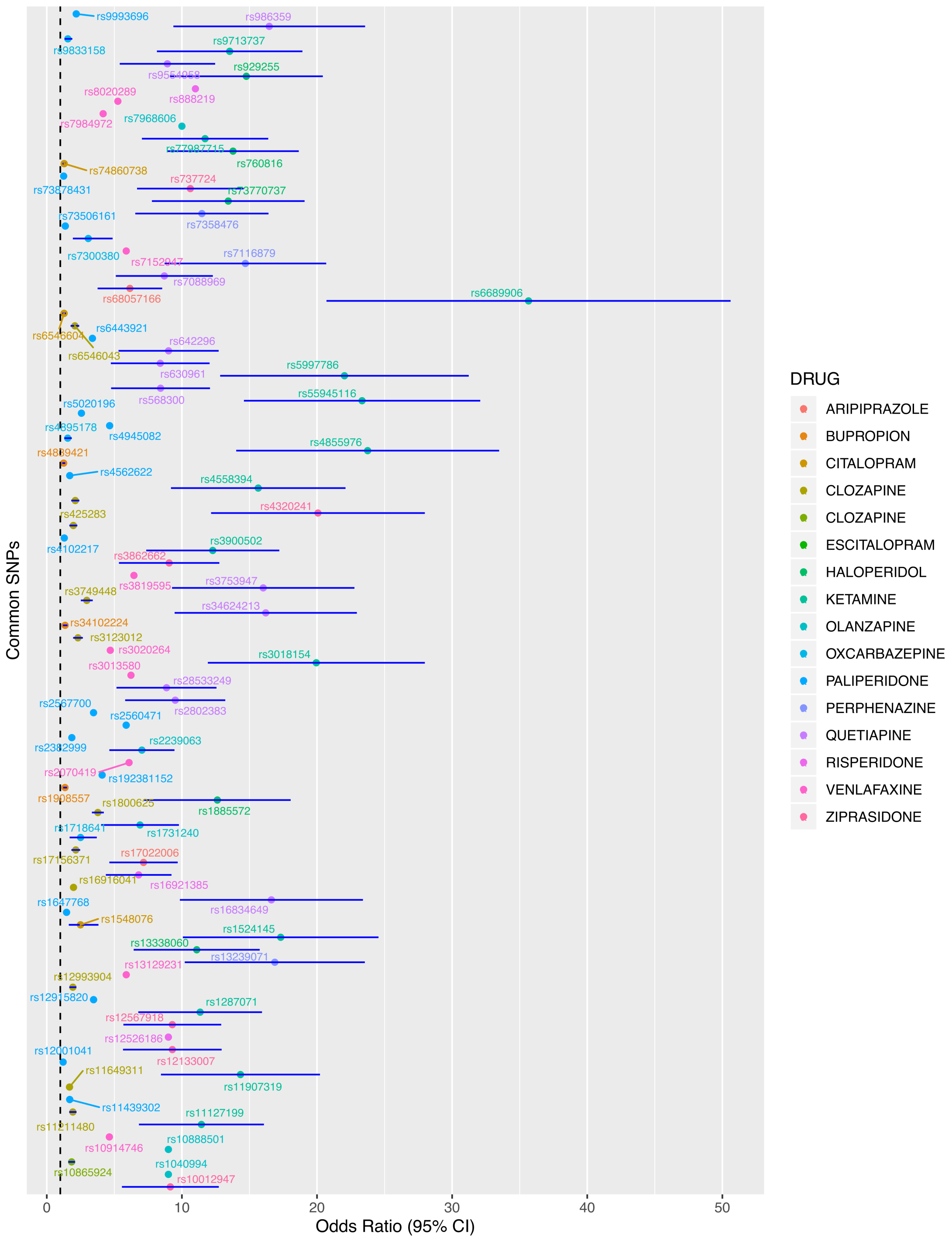

Figure S2. Odds ratio values (between 1-60) of pharmacogenomics (PGx) variants of common frequency (MAF > 0.10) are plotted alongside with the 95% of their confidence intervals. The PGx variants are colored based on the antipsychotic or antidepressant drug of the association.

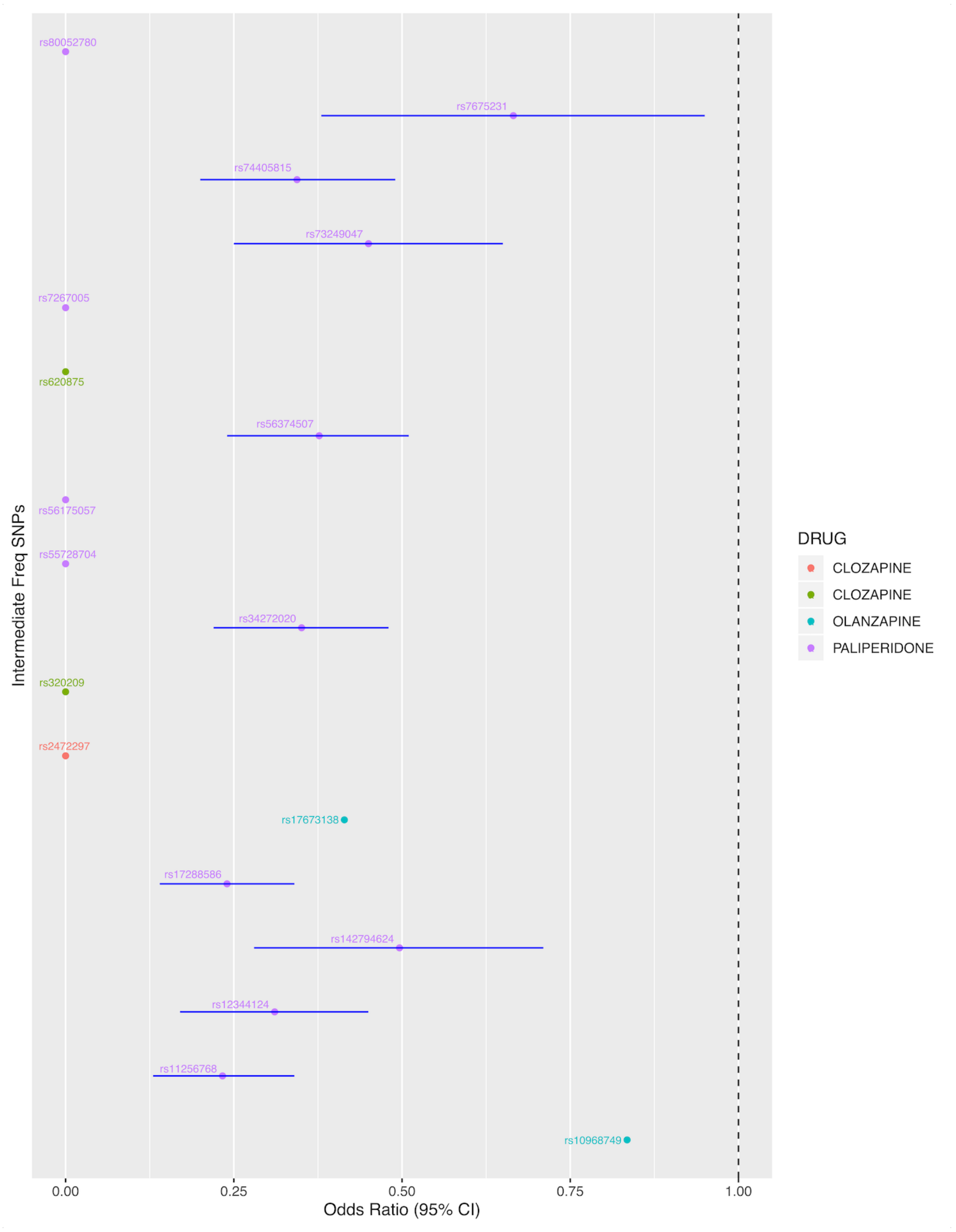

Figure S3. Odds ratio values (between 0-1) of pharmacogenomics (PGx) variants of intermediate frequency (0.05 < MAF < 0.10) are plotted alongside with the 95% of their confidence intervals. The PGx variants are colored based on the antipsychotic or antidepressant drug of the association. Abbreviations: Intermediate Freq, intermediate frequency.

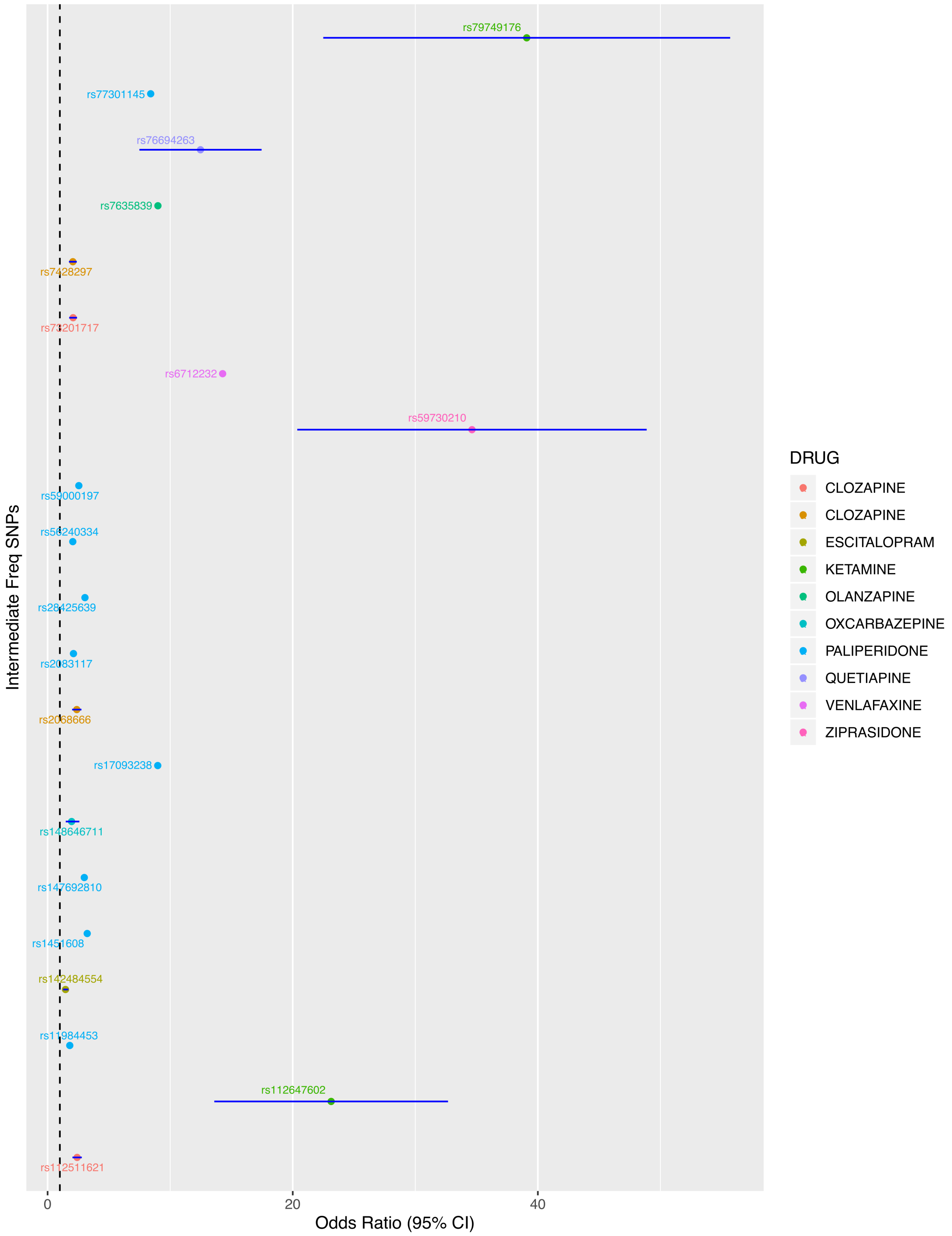

Figure S4. Odds ratio values (between 1-60) of pharmacogenomics (PGx) variants of intermediate frequency (0.05 < MAF < 0.10) are plotted alongside with the 95% of their confidence intervals. The PGx variants are colored based on the antipsychotic or antidepressant drug of the association. Abbreviations: Intermediate Freq, intermediate frequency.

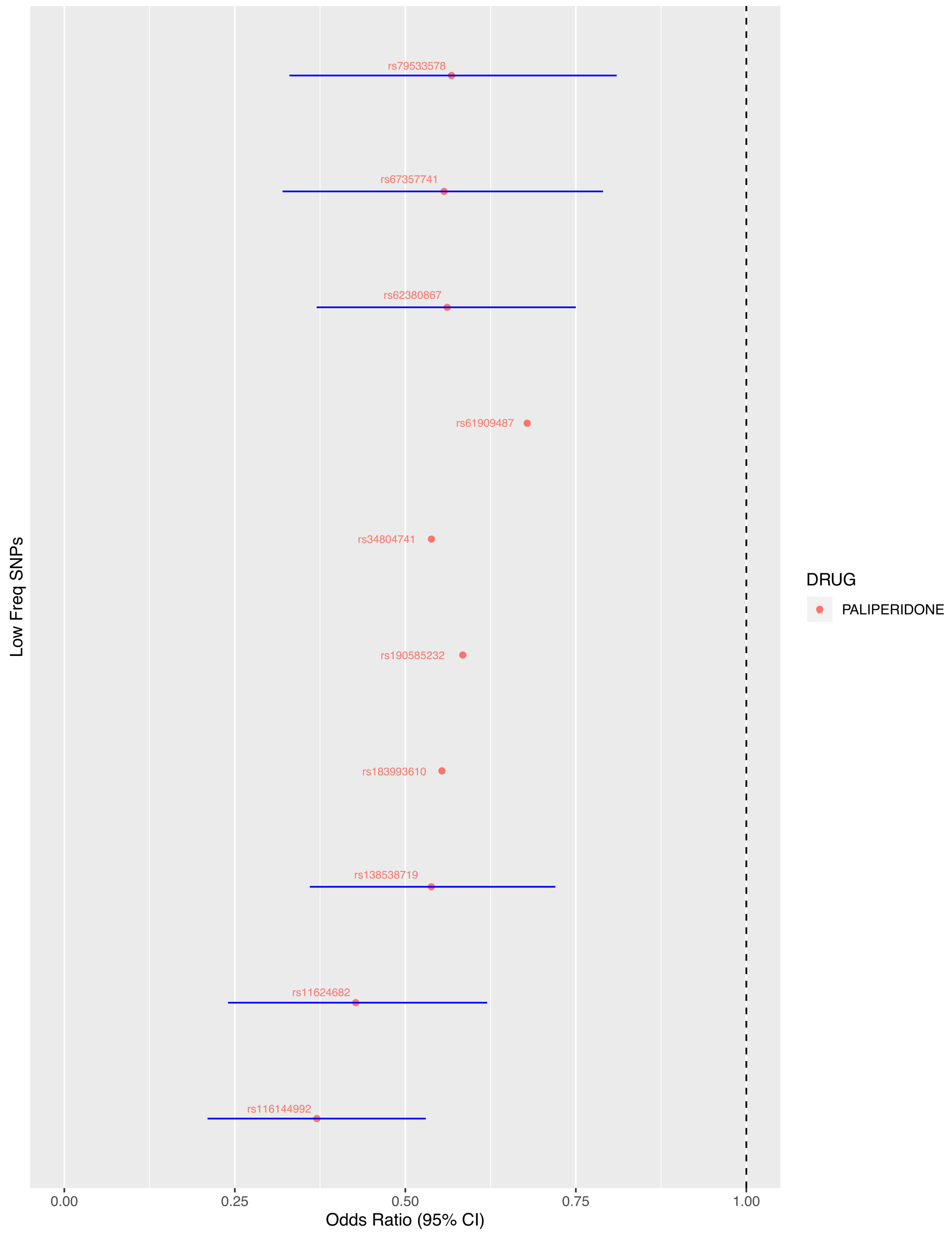

Figure S5. Odds ratio values (between 0-1) of pharmacogenomics (PGx) variants of low frequency (0.05 < MAF < 0.10) are plotted alongside with the 95% of their confidence intervals. The PGx variants are colored based on the antipsychotic or antidepressant drug of the association. Abbreviations: Low Freq, low frequency.

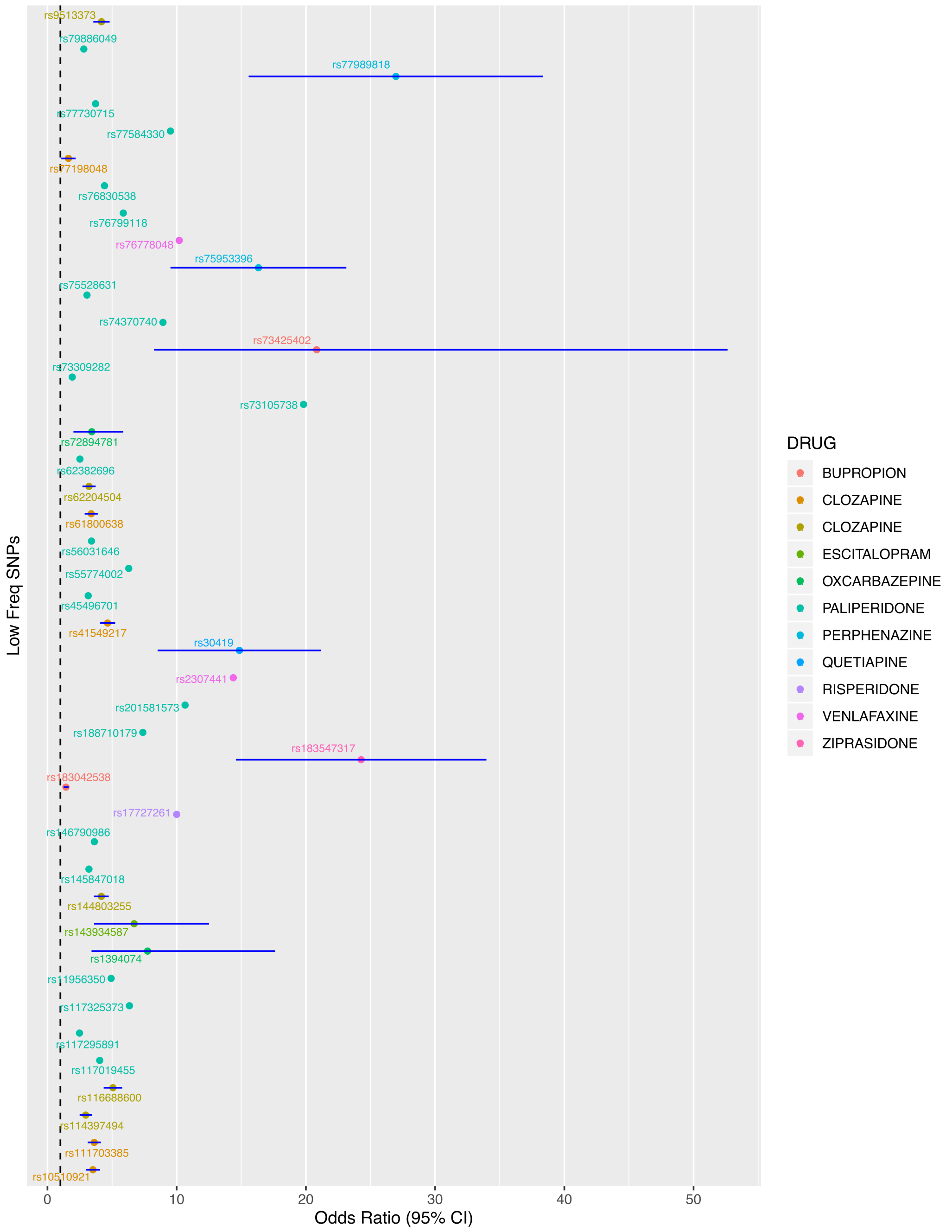

Figure S6. Odds ratio values (between 1-60) of pharmacogenomics (PGx) variants of low frequency (0.05 < MAF < 0.10) are plotted alongside with the 95% of their confidence intervals. The PGx variants are colored based on the antipsychotic or antidepressant drug of the association. Abbreviations: Low Freq, low frequency.

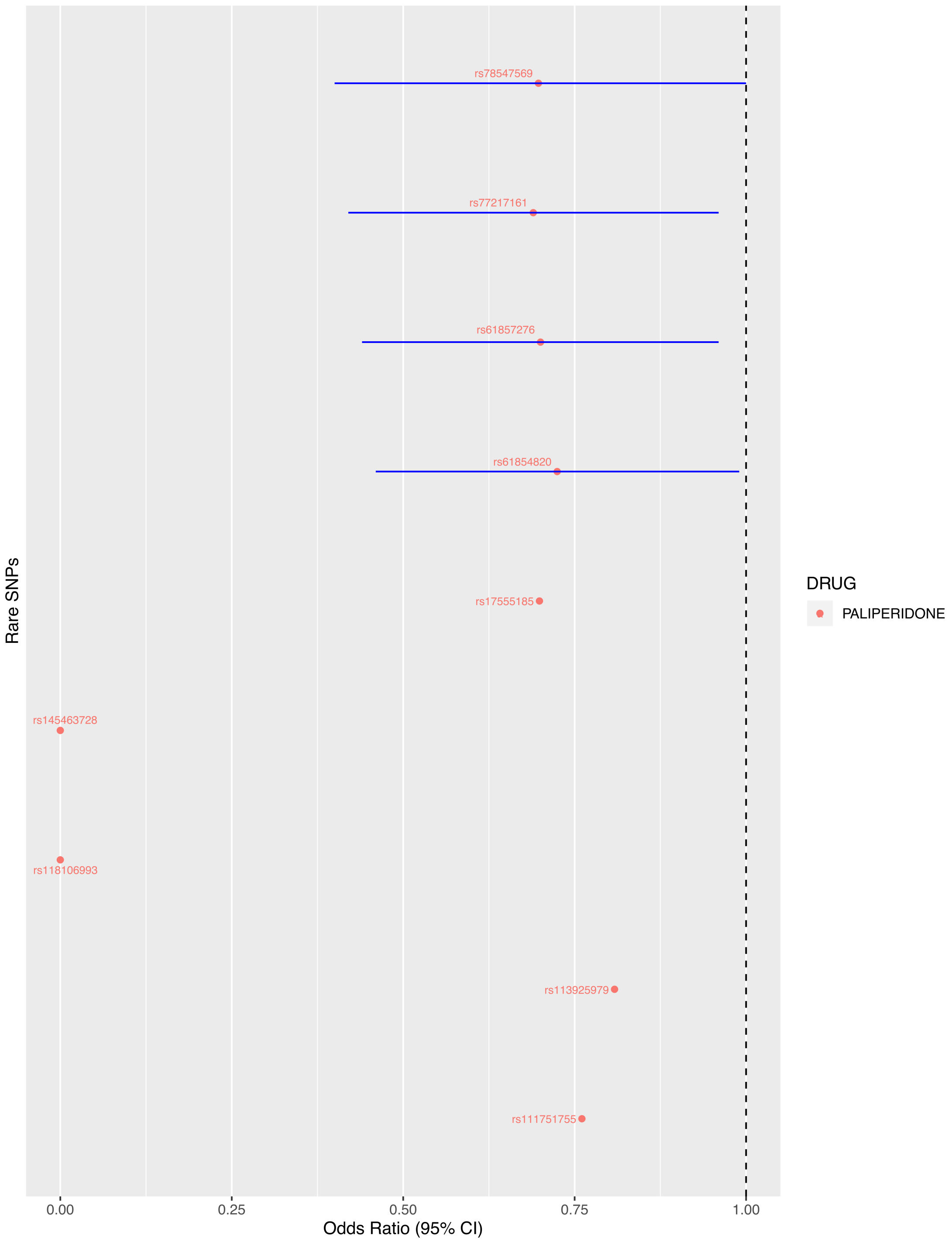

Figure S7. Odds ratio values (between 0-1) of pharmacogenomics (PGx) variants of rare frequency (0.05 < MAF < 0.10) are plotted alongside with the 95% of their confidence intervals. The PGx variants are colored based on the antipsychotic or antidepressant drug of the association.

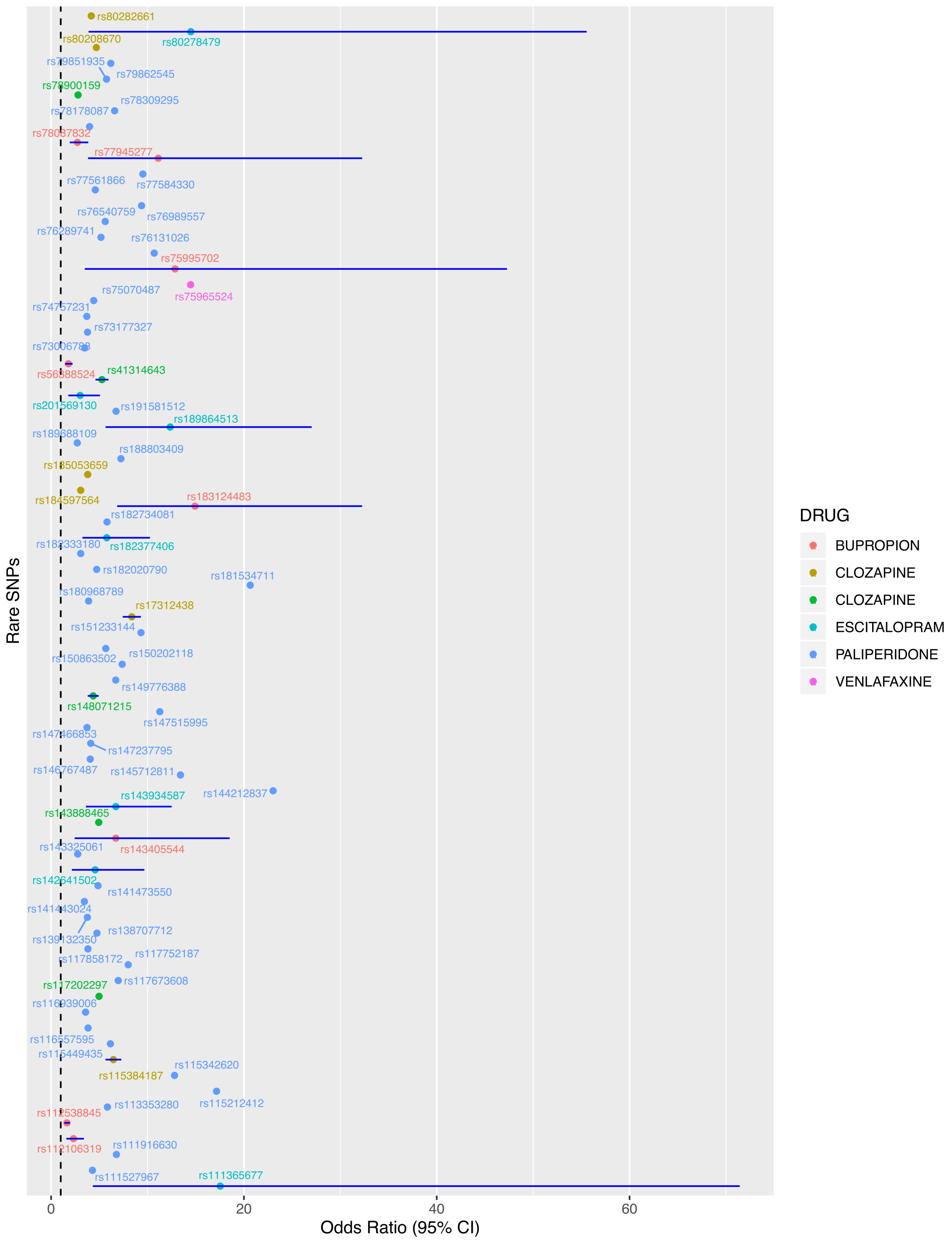

Figure S8. Odds ratio values (between 1-60) of pharmacogenomics (PGx) variants of rare frequency (0.05 < MAF < 0.10) are plotted alongside with the 95% of their confidence intervals. The PGx variants are colored based on the antipsychotic or antidepressant drug of the association.

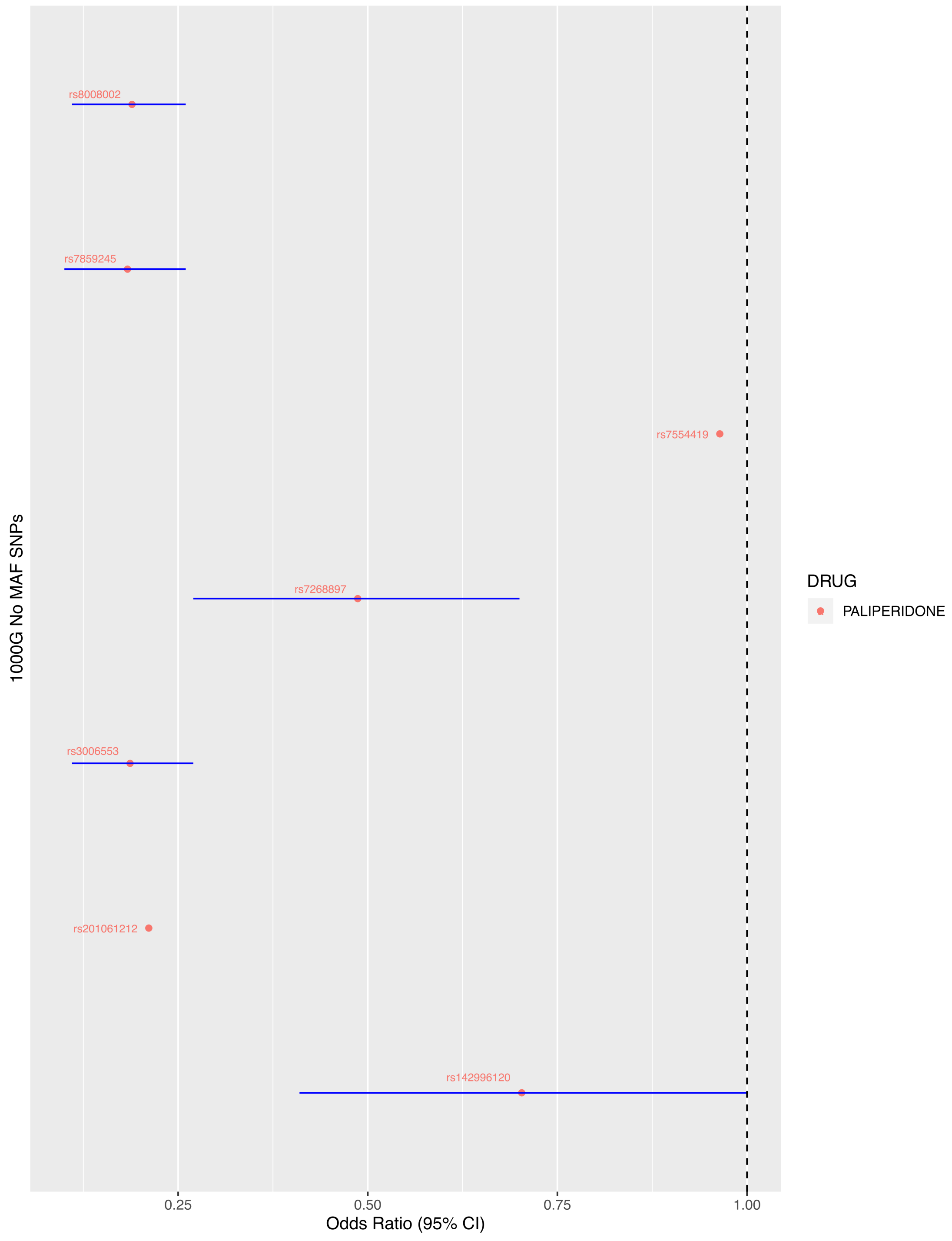

Figure S9. Odds ratio values (between 0-1) of pharmacogenomics (PGx) variants, which were not found in 1000Genomes Project, are plotted alongside with the 95% of their confidence intervals. The PGx variants are colored based on the antipsychotic or antidepressant drug of the association. Abbreviations: 1000G No MAF, not found in 1000Genomes Project (no MAF was found).

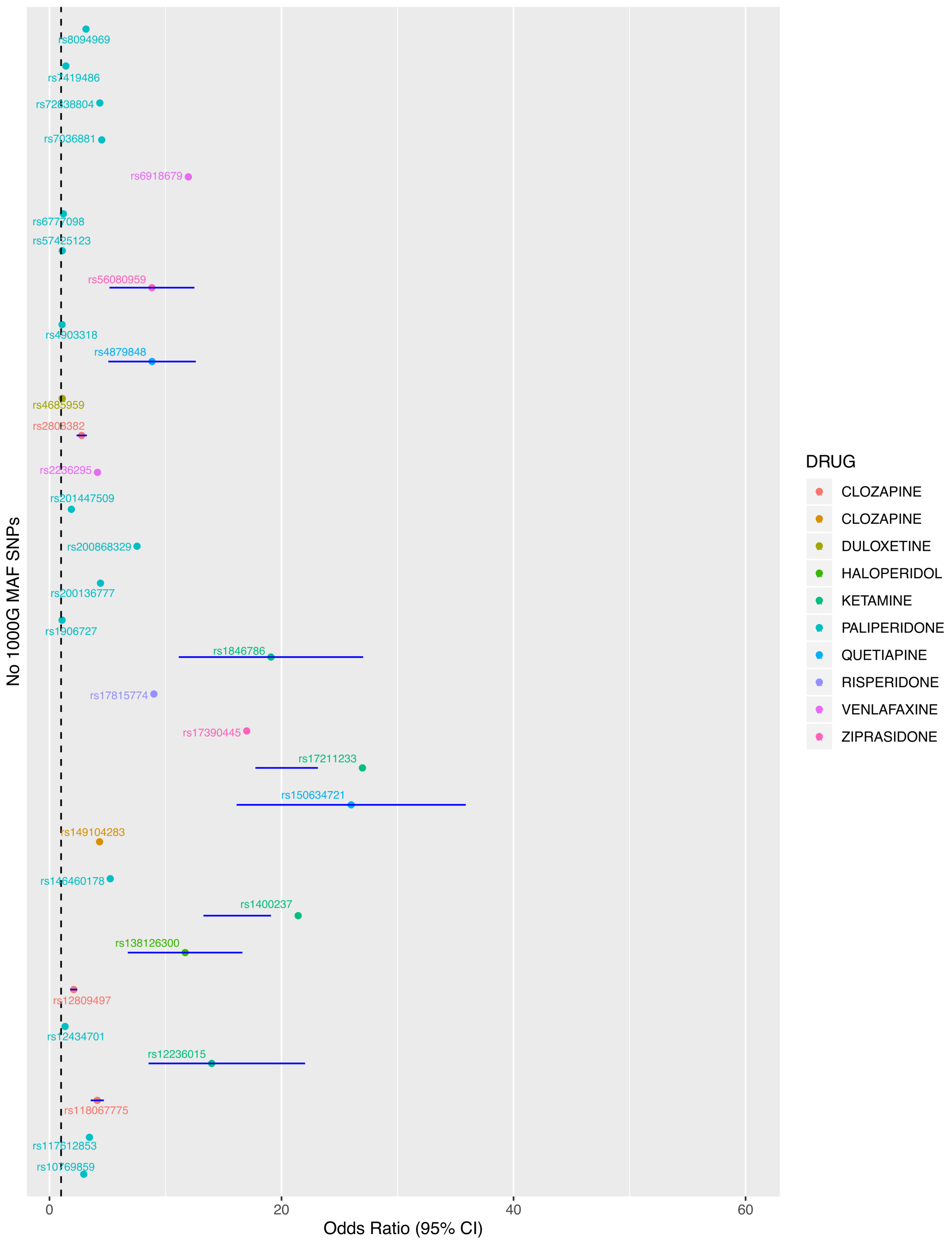

Figure S10. Odds ratio values (between 1-60) of pharmacogenomics (PGx) variants, which were not found in 1000Genomes Project, are plotted alongside with the 95% of their confidence intervals. The PGx variants are colored based on the antipsychotic or antidepressant drug of the association. Abbreviations: 1000G No MAF, not found in 1000Genomes Project (no MAF was found).
